# Accounting Transparency, Fear Sentiment and the COVID-19 Epidemic: For Public Health Security and the Construction of an Early Warning System

**DOI:** 10.3389/fpubh.2022.908430

**Published:** 2022-07-19

**Authors:** Haiyan Wang, Min Sun, Han Li, Diantong Kang, Lei Yan, Jianhao Gao

**Affiliations:** ^1^Business School, Zhejiang Wanli University, Ningbo, China; ^2^Finance Office, Gansu Provincial Hospital of TCM, Lanzhou, China; ^3^School of Mathematics and Statistics, Hexi University, Zhangye, China

**Keywords:** fear sentiment, accounting transparency, COVID-19, blockchain, public health, public health security early warning system

## Abstract

A central issue of public health security and the construction of an early warning system is to establish a set of responsibility-oriented incentives and restraint mechanisms. This is closely related to the accounting transparency of the institutional environment and the fear sentiment of the individual's predicament. This study analyses the relationship between accounting transparency, fear sentiment, and COVID-19 through a VAR model analysis. The results show a significant and negative relationship between accounting transparency and daily new COVID-19 patients. In particular, accounting transparency has a negative impact on the increase in the number of people infected with a two-period lag, while the three-period lag in the number of new epidemics has a negative impact on accounting information. Second, accounting transparency has a positive impact on the increase in the search volume on COVID-19 within a three-period lag. After the three-period lag, the number of new epidemics has a positive impact on accounting information. Third, an increase in fear sentiment can be driven by the fear of COVID-19. Fourth, in the public health early warning system, according to the abovementioned time characteristics, the system arranges the emotional counseling, early warning incentives, and institutional constraints to be dealt with in the first 4 days. In addition, in the early warning target-oriented system setting, the parallel system helps to improve the early warning efficiency.

## Introduction

For a period of time after the outbreak of the COVID-19 epidemic, considerable literature has grown up around the theme of public health security. Although there are over 200,000 publications related to the many aspects of COVID-19, as of August 2021 (1), it was difficult for society to trace the source of the initial COVID-19 infection. This is because, on the one hand, after the outbreak of the COVID-19 epidemic, society paid more attention to medical and health emergency management (2) while ignoring early warning signs. On the other hand, due to people's concerns with privacy, traceability causes some people social disgust (3). In addition, the increase in the number of asymptomatic infections and the difficulty of screening (4), as well as the recovery of the economy (5, 6), have caused us to forget the importance of early warning. Lu et al. showed that genetically, SARS-CoV-2 (mainly COVID-19) is 79% identical to SARS-CoV and 50% identical to MERS-CoV (7). This study stresses the importance of early warning of epidemics. How can the Public Health Security Early Warning System be constructed? The difficulty in answering this question is not just technical, but also because it is difficult to trace the origin of the merchants. From a technical point of view, existing smartphone-based GPS and social media technology may provide a suitable case, which can help us to trace the close contacts of all patients (8). From the perspective of economics, in existing research, Google search volume (9–11) and Baidu search volume (12) provide a method for us to understand the fear sentiment of the public (13). From the perspective of accounting information, the purpose of accounting is to provide useful information for decision-making to major stakeholders outside the enterprise. The higher the accounting transparency is, the more helpful it is to improve the efficiency of resource allocation. In turn, transaction costs can be reduced, and a contractual ex post resolution mechanism can be formed (14). However, this idea has not been applied to early warning, and it is necessary to conduct special research on it.

The COVID-19 epidemic has made the public security of some countries rise to national security (15, 16). In reality, due to the difficulty in determining where the source of the disease came from and where it is going, public health security management becomes difficult. However, it is still necessary to construct a public health security early warning system (17). Generally, a public health security early warning system requires two factors. On the one hand, with the construction of the institutional environment, it is important to build a governance mechanism with clear full responsibility (18). In terms of measuring the variable of accounting transparency (institutional environment), the Opacity Index and Corruption Perceptions Index can provide useful exploration. The Opacity index aims to share that greater transparency across many dimensions of capital markets, and encourages investor confidence and keeps the costs of doing business under control (14, 19, 20). It is designed by the PricewaterhouseCoopers Endowment for the Study of Transparency and Sustainability and is based on data from five different areas—corruption, legal systems, government macroeconomic and fiscal policies, accounting standards, and practices (including corporate governance and information release), and regulatory regimes. The Corruption Perception Index was published by Transparency International. The scores range from 10 (highly clean) to 0 (highly corrupt), and 102 countries were evaluated. The ranking was determined by 15 different surveys from nine independent institutions (21–23). From an individual point of view, fear sentiment arises during the infection process of the new coronavirus epidemic. Current research in this area mostly focuses on the types and characteristics of emotions (24, 25). In fact, since this emotional feature is closely related to life, we must not only avoid inappropriate reporting that may exacerbate panic but also channel this panic and form an institutional mechanism for early warning. Considering accounting transparency and fear sentiment, it would be helpful to build a public health security early warning system. The specific element setting and element organization must coincide. Briefly, among the five safety factors of human, material, technology, management, and environmental factors, we should focus on human factors, such as doctors, disease control, scientific research, administration, citizens, businessmen, etc. In addition, in terms of the organization of elements, it is possible to analyze the required element configuration and technical configuration, starting from the reliability of the “serial” system, which is not as good as the “parallel” system (26).

The paper's contributions are as follows: first, during the COVID-19 epidemic, it is difficult to judge whether accounting transparency is effective for epidemic control. Therefore, we creatively use two proxy variables to complete this logic. A proxy of fear sentiment induced by COVID-19 using high-frequency Google search data (11). Another proxy of accounting transparency using the ratio of stock market capitalization divided by GDP has a statistically significant relationship with the opacity index (27). The results imply that our variable selection is appropriate by cleverly exploiting their correlation with the stock market. Second, we used epidemic data from many countries to verify the robustness of our findings. Such countries include the United States, China, the United Kingdom, Japan, Italy, Spain, Germany, France, Iran, South Korea, Canada, Australia, and Singapore. Third, we have clearly explained that accounting transparency can reduce market fears, which has significance for epidemic prevention and control. In the construction of the early warning system, taking advantage of the characteristics of market fear and adopting a parallel system will enable better prevention and control of the epidemic.

The outline of the remainder of the paper is as follows: In Section Literature Review, the current research and literature in the field are reviewed. In Section Data and Empirical Analysis, the data and process of empirical analysis are explained. Section Further Discussion: Organization of Public Health Security and an Early Warning System discusses the details and organization of the public health security early warning system. Finally, the conclusion elaborates on the empirical results.

## Literature Review

Historically, some research in the institutional environment has focused on the Opacity Index. Some researchers have proposed the impact of accounting transparency on economic growth (28). Wei) supports that transparency lowers the effective rate of the “corruption tax” (29). Stultz describes that opacity inhibits the ability of corporate governance systems to overcome informational asymmetry because the cost of capital can be affected by opacity in several ways (30). Kamiru and McGowanused the opacity index for 2005/2006, 2007/2008, and 2009 and found a statistically significant relationship between the opacity index and the ratio of stock market capitalization divided by GDP for a sample of 45 countries (27). To date, several studies have used the corruption perceptions index to characterize the institutional environment (21–23, 28, 29, 31). In terms of fear sentiment, several studies have shown an increase in the prevalence of anxiety and depression symptoms during the COVID-19 outbreak (25, 32–36). Jarrett et al. proposed that mental health disorders have increased during the pandemic through data from European and Southeast Asian countries (37). The internet high-frequency search data provided a method for us to measure the public's fear, such as Google search volume and Baidu search volume (9–12).

The above studies provide a logical basis for the construction of a public health security early warning system. In terms of mechanisms of the early warning system, it is recommended to integrate the responsibilities and interests of the relevant personnel. Häsler et al. integrated nutrition, food safety, and value chain analysis into a comprehensive framework to analyse food safety issues in dairy value chains in two regions of Tanzania through rural participatory assessments (38). In the organization of the early warning system, Jongejan et al. mathematically deduced that by designing the safety chain as a “parallel” system, its reliability will be much higher than that of a “series” system (26). Hazards may be physical, man-made or naturally occurring chemicals or organisms or viruses that reproduce within a specified life cycle. Blockchain technology originates from the Byzantine Generals problem that solves information asymmetry. In the technology of early warning system construction, Saito proposed that blockchain is a set of database technologies designed to create a digital currency system represented by Bitcoin (39). Blockchain has the characteristics of decentralization, trustlessness, traceability, and immutability, which makes the use of the internet possible to change from traditional information transmission to value transmission (40). Through extensive consensus and value sharing, new rules and value systems can monitor and automatically warn the detection data of the entire supply chain in real time.

The literature on accounting transparency and fear sentiment form a relationship that is a combination of the institutional environment and individual characteristics. The literature on the construction of a public health security early warning system suggests that the traditional approach might be less effective when the epidemic occurs. We can see that the risk prevention and control of public health security have their own laws, and it is necessary to treat risk prevention and control and risk assessment as one. A parallel-based safety chain is the organization's risk management for low-probability disasters, and public health security should pay attention to an analysis of system reliability. The distributed and immutable characteristics of blockchain technology help to build a warning system for public health security.

## Data and Empirical Analysis

### Background

**Figure 1:**
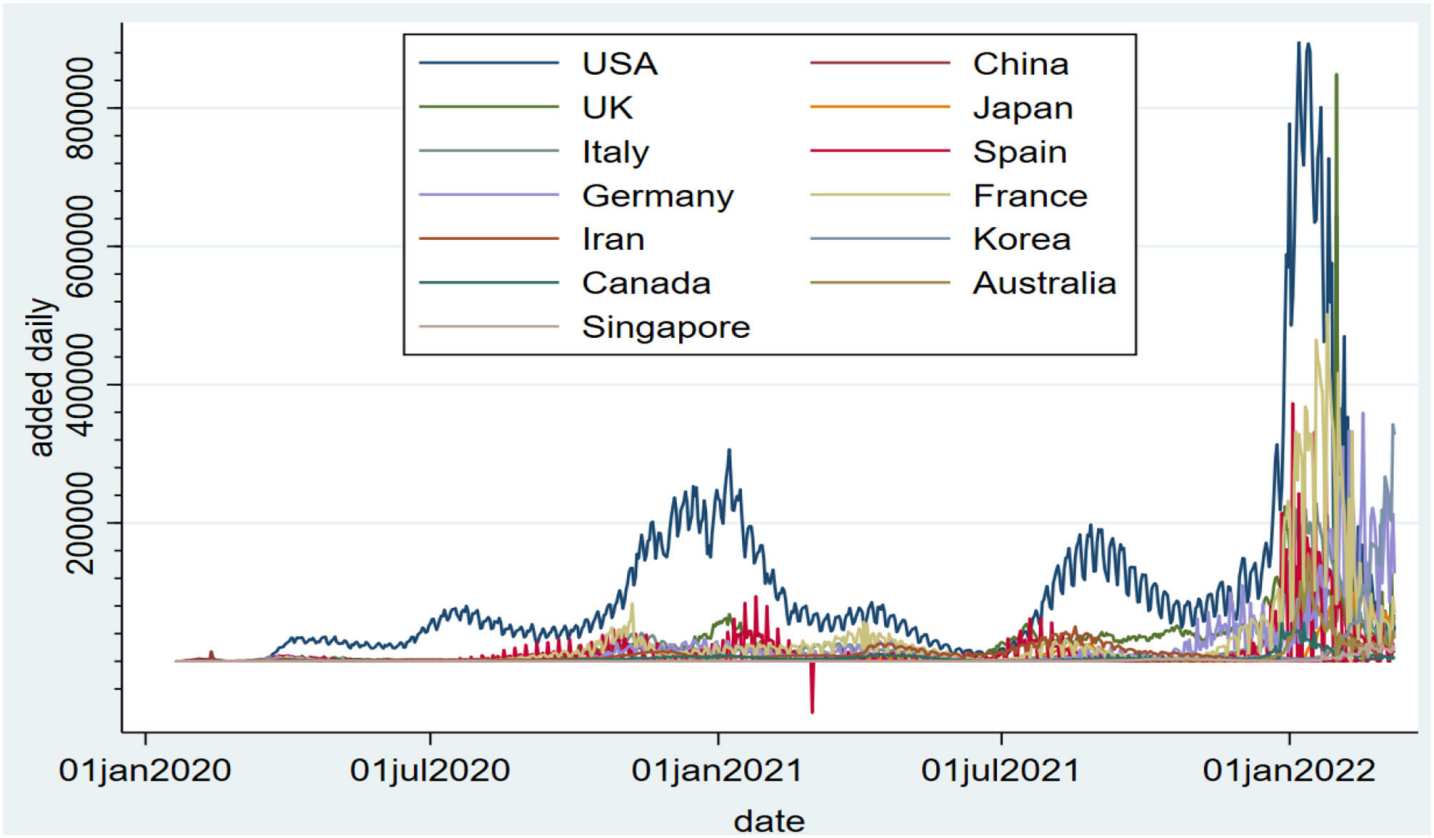
COVID-19 daily value added.

Judging from the daily increase in epidemics, an important way for China to deal with emergencies, such as public health is through joint prevention and control, including an early warning system based on a parallel approach. The effectiveness of joint prevention and control in this epidemic has been greatly understood. Judging from the daily increase in COVID-19 epidemics in the United States, China, the United Kingdom, Japan, Italy, Spain, Germany, France, Iran, South Korea, Canada, Australia, and Singapore, China has had the fastest control of the epidemic and has maintained zero growth for an extended period of time. Refer to Figure 1 for details. In March 2021, the number of people vaccinated per 100 people in the UK was 30.17, and by June 15, 2021, this figure showed a steady increase, reaching 61.9. However, the epidemic in the UK has not been effectively controlled: it has rebounded since mid-to-late May, and the number of newly diagnosed patients has increased sharply. The number of new cases reached 7,920 on June 12. The epidemic prevention and control in the UK has not achieved the expected results. As of June 16, 2021, the number of people vaccinated per 100 people in the United States was 93.56. Vaccination has alleviated the epidemic situation in the United States to a certain extent. However, due to unsatisfactory prevention and control, the number of new people per day is still high even at a high vaccination rate. By comparing the daily new correlation coefficients (see Table 1 for details, the lower-left corner is the Pearson correlation coefficient, and the upper right corner is the Spearman correlation coefficient), in terms of the daily number of new COVID-19 patients, the relationship between each country and the global new epidemic situation in both correlation coefficients, China's value is very low. At the same time, the data related to the daily new epidemics in other countries shows that the daily new epidemics in China and other countries are also very low. In comparison, China's joint prevention and control method has certain advantages in epidemic prevention and control.

**Table 1:** Correlation coefficient.

	**Global**	**China**	**USA**	**Italy**	**Spain**	**Germany**	**France**	**UK**	**Iran**	**Korea**	**Japan**	**Canada**	**Singapore**
Global	1	0.250^***^	0.751^***^	0.818^***^	0.379^***^	0.845^***^	0.846^***^	0.706^***^	0.651^***^	0.804^***^	0.782^***^	0.828^***^	0.229^***^
China	0.155^***^	1	0.113^***^	0.148^***^	0.023	0.177^***^	0.102^***^	0.200^***^	0.012	0.347^***^	0.276^***^	0.101^***^	0.037
USA	0.806^***^	−0.067*	1	0.708^***^	0.326^***^	0.732^***^	0.669^***^	0.738^***^	0.414^***^	0.590^***^	0.543^***^	0.721^***^	0.250^***^
Italy	0.897^***^	0.077^**^	0.863^***^	1	0.323^***^	0.902^***^	0.890^***^	0.660^***^	0.427^***^	0.620^***^	0.572^***^	0.846^***^	0.085^**^
Spain	0.623^***^	0.009	0.634^***^	0.543^***^	1	0.352^***^	0.302^***^	0.335^***^	0.241^***^	0.243^***^	0.281^***^	0.343^***^	0.039
Germany	0.762^***^	0.364^***^	0.383^***^	0.579^***^	0.320^***^	1	0.822^***^	0.716^***^	0.455^***^	0.728^***^	0.535^***^	0.864^***^	0.286^***^
France	0.891^***^	0.044	0.794^***^	0.952^***^	0.505^***^	0.586^***^	1	0.647^***^	0.473^***^	0.655^***^	0.610^***^	0.735^***^	0.121^***^
UK	0.643^***^	0.035	0.675^***^	0.562^***^	0.543^***^	0.428^***^	0.527^***^	1	0.446^***^	0.771^***^	0.470^***^	0.639^***^	0.389^***^
Iran	0.315^***^	−0.050	0.092^**^	0.055	0.072^**^	0.240^***^	0.100^***^	0.153^***^	1	0.587^***^	0.739^***^	0.467^***^	0.063*
Korea	0.352^***^	0.789^***^	0.004	0.201^***^	0.080^**^	0.641^***^	0.161^***^	0.145^***^	0.079^**^	1	0.586^***^	0.607^***^	0.413^***^
Japan	0.741^***^	0.376^***^	0.312^***^	0.523^***^	0.280^***^	0.857^***^	0.562^***^	0.334^***^	0.432^***^	0.634^***^	1	0.604^***^	0.108^***^
Canada	0.727^***^	0.009	0.833^***^	0.773^***^	0.699^***^	0.377^***^	0.677^***^	0.640^***^	0.046	0.087^**^	0.263^***^	1	0.179^***^
Singapore	0.479^***^	0.588^***^	0.082^**^	0.296^***^	0.111^***^	0.762^***^	0.281^***^	0.221^***^	0.162^***^	0.866^***^	0.782^***^	0.129^***^	1

*, **, *** *show 10%, 5% and 1% level significance levels respectively*.

**Figure 2:**
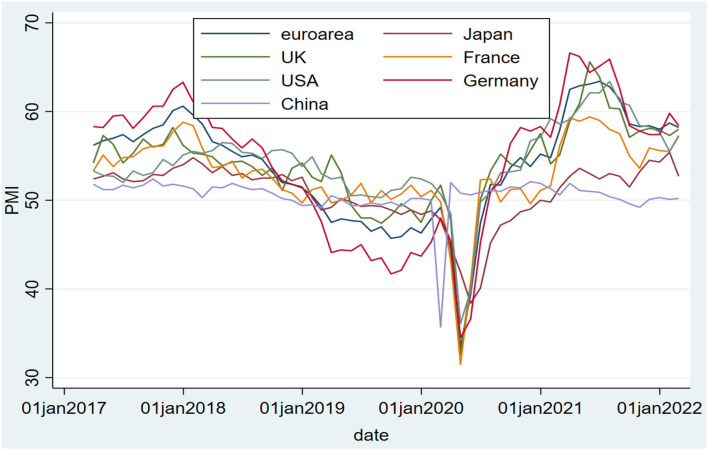
Manufacturing PMI values for several countries.

From an economic perspective, in 2020, when the epidemic broke out, the year-to-year ratios of China's third and fourth quarters of 2019 were 6 and 5.8, respectively. Under the action of the joint prevention and control mechanism, growth was expected to resume in the second quarter of 2020 and by the fourth quarter of 2021, and GDP (gross domestic product) would maintain year-on-year growth. In contrast, countries or regions with other epidemic prevention measures have been affected more. For example, in the European Union, the year-on-year ratios in the third and fourth quarters of 2019 were 1.7 and 1.2, respectively. In the first quarter of 2020, when the epidemic broke out, GDP fell to −3.1 year-on-year. In the second quarter, when the epidemic was at its worst, it fell to −14.5, with growth not returning until Q2 2021. By analyzing the CPI (consumer price index), we find that China's CPI rose sharply during the worst period of the epidemic, peaking in January at 5.4. Under the action of the joint prevention and control mechanism, with the control of the epidemic, the CPI began to gradually decline and return to normal levels. At the same time, U.S. epidemic prevention and control strategies have not achieved ideal results. As of January 2022, the CPI has reached 7.5, facing severe inflation. There is a similar problem in the United Kingdom, which has been rising since March 2021, and the CPI was expected to reach 5.4 in January 2022. By analyzing the manufacturing PMI (Purchasing Managers' Index), we found that China's PMI index was near the line of prosperity and declined after the epidemic, and the change was not large. This is because, with the help of joint prevention and control, China has completed the initial strong economic rebound after the epidemic. The stage is now slowly entering normalization, and the PMI is now slowly falling back to near the line of prosperity and decline. By comparing the manufacturing PMIs of the European Union, Japan, the United Kingdom, France, the United States, Germany, and China, those countries with a better industrial base have a better degree of economic recovery than China after the coronavirus epidemic. Please refer to Figure 2 for details.

### Data and Variables

#### Accounting Transparency

Referring to the opacity index (OPI) for 2005/2006, 2007/2008, and 2009 (19, 20), we based our analysis on the research hypothesis that there is a statistically significant relationship between the Opacity Index and the ratio of stock market capitalization divided by GDP (27). This study used an instrumental variable, that is, the ratio of the daily total market capitalization of listed companies to GDP. This variable characterizes the institutional environment of information. The data used was sourced from Wind.

#### Fear Sentiment

To measure fear sentiment about the COVID-19 pandemic, we used data from Google Trends based on internet searches, which is consistent with previous research (41). In addition, the VIX was also used to reflect fear sentiment (42). The VIX was calculated based on real-time S&P 500 call and put options. We used this approach to test the robustness of the results.

#### Daily New COVID-19 Patients

This data reflects the daily increase in COVID-19 epidemics from 20 January 2020 to 9 March 2022, including global data from the United States, China, Japan, Korea, Spain, the United Kingdom, Australia, France, Singapore, Italy, Germany, Canada, and other countries. This data came from the Wind database, and the data from Japan and other countries were mainly tested for robustness.

Preliminary analysis Tables 2, 3 report descriptive statistics and correlation coefficients for the data used in this study, including accounting transparency, fear sentiment, and daily new COVID-19 patients (global). The fear sentiment has a minimum value of zero from 20 January 2020 to 16 February 2020, which indicates that COVID-19 has not received attention. From good correlation coefficient values, possible regression between accounting transparency, fear sentiment, and the daily new COVID-19 patients (global) was expected.

**Table 2:** Descriptive statistics.

**Variable**	**Obs**	**Mean**	**Std. Dev**.	**Min**	**Max**
Lntransparency	780	1.017	0.201	0.431	1.312
lnGlobal	780	12.58	1.65	4.898	15.184
lnSearchpopularity	753	3.885	0.548	0	4.605

**Table 3:** Correlation coefficient.

	**lnusaopacity**	**lnGlobal**	**lnSearchpopularity**
Lntransparency	1	0.763***	0.121^***^
lnGlobal	0.715^***^	1	0.411^***^
lnSearchpopularity	0.212^***^	0.714^***^	1

*, **, *** *show 10%, 5% and 1% level significance levels respectively*.

### Method: VAR

Following Urquhart (43) and Chen et al. (11), vector autoregressive (VAR) models were used to examine the relationship between accounting transparency, fear sentiment, and daily new COVID-19 patients (global). A VAR was used to capture the dynamics of multiple time series. Tantaopas et al. (44) proposed it as an appropriate estimation technique to examine the interdependence of dynamic relationships. In my model selection, the time-varying parameter vector autoregressive model (TVP-VAR) is an extension of the VAR model. Its biggest improvement lies in the assumption that both the coefficient matrix and the covariance matrix are time-varying, which is beneficial to characterize the non-linear characteristics of the simultaneous relationship between variables. We used the TVP-VAR model, but the model did not work well. So we still used the traditional VAR model.

Let *X*_t_ be a vector of variables of interest; then, a VAR (*p*) model will have the following structure:


$$\begin{array}{r} X_{t}=\alpha +\overset{p}{\underset{j=1}{\sum }}\beta_{j}X_{t-j}+\varepsilon_{t} \end{array}$$

where α is a vector of constants, β_*j*_ is a vector of coefficients, and ε_*t*_ is a vector of independent white noise innovations. *p* denotes the number of optimal lags determined by several information criteria.

### Empirical Results

**Figure 3:**
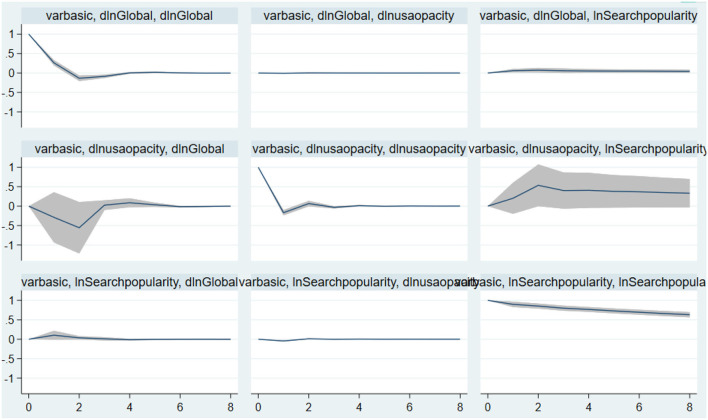
Structural impulse response.

We performed a unit root test on accounting transparency, fear sentiment, and the log difference of the daily new COVID-19 patients (global), and all passed the test. Then, we analyzed the optimal lag order, as shown in Table 4. The largest lag order was 4. The model was then estimated. The coefficients and Granger causality test results are shown in Table 5. The graph of the structural impulse response is shown in Figure 3.

**Table 4:** Lagtest.

**Lag**	**LL**	**LR**	**df**	**p**	**FPE**	**AIC**	**HQIC**	**SBIC**
0	2,055.490	0.000	−5.481	−5.473	−5.462			
1	3,416.940	2,722.900	9	0.000	0.000	−9.092	−9.063	−9.018
2	3,467.280	100.680	9	0.000	0.000	−9.202	−9.152	−9.073
3	3,525.750	116.950	9	0.000	0.000	−9.334	−9.263	−9.149
4	3,577.820	104.13^*^	9	0.000	1.6e-08^*^	−9.44944*	−9.35677^*^	−9.20895^*^

* *Show 10% level significance levels*.

**Table 5:** VAR estimations for accounting transparency, fear sentiment, and global daily new patient.

	**Coef**.	**Std. Err**.	***z***	***P* > *z***	**(95%Conf.)**	**(Interval)**
**dlntransparency**						
**lnSearchpopularity**						
L1.	−0.045	0.006	−6.950	0.000	−0.057	−0.032
L2.	0.054	0.009	6.230	0.000	0.037	0.071
L3.	−0.023	0.009	−2.680	0.007	−0.041	−0.006
L4.	0.015	0.006	2.460	0.014	0.003	0.028
**dlnGlobal**						
L1.	−0.007	0.004	−1.750	0.081	−0.015	0.001
L2.	0.009	0.004	2.260	0.024	0.001	0.017
L3.	−0.009	0.004	−2.260	0.024	−0.017	−0.001
L4.	0.005	0.004	1.130	0.260	−0.003	0.013
_cons	−0.004	0.004	−1.000	0.320	−0.012	0.004
**lnSearchpopularity**						
**Dlntransparency**						
L1.	0.177	0.206	0.860	0.391	−0.227	0.580
L2.	0.216	0.207	1.040	0.297	−0.190	0.623
L3.	−0.901	0.203	−4.450	0.000	−1.298	−0.504
L4.	−0.056	0.202	−0.280	0.781	−0.453	0.340
dlnGlobal
L1.	0.076	0.023	3.320	0.001	0.031	0.121
L2.	−0.014	0.023	−0.610	0.543	−0.058	0.030
L3.	0.079	0.023	3.490	0.000	0.035	0.123
L4.	0.017	0.023	0.710	0.477	−0.029	0.063
_cons	0.247	0.024	10.330	0.000	0.200	0.293
**dlnGlobal**						
**lntransparency**						
L1.	−0.283	0.303	−0.930	0.351	−0.877	0.311
L2.	−0.277	0.305	−0.910	0.365	−0.876	0.322
L3.	−0.916	0.298	−3.070	0.002	−1.500	−0.331
L4.	−0.582	0.298	−1.950	0.051	−1.165	0.002
**dlnGlobal**						
L1.	0.098	0.034	2.900	0.004	0.032	0.164
L2.	−0.183	0.033	−5.500	0.000	−0.248	−0.118
L3.	−0.235	0.033	−7.060	0.000	−0.300	−0.169
L4.	−0.301	0.035	−8.720	0.000	−0.369	−0.233
_cons	−0.036	0.035	−1.010	0.311	−0.105	0.033

#### Accounting Transparency and COVID-19

As expected, we find that a decrease in daily new COVID-19 patients can be driven by accounting transparency, as indicated by a significant and negative relationship between accounting transparency and daily new COVID-19 patients. In particular, accounting transparency has a negative impact on the increase in the number of people infected with a two-period lag. That is, after a two-day lag, with the release of information, the number of people infected was brought under control. At the same time, the three-period lag in the number of new epidemics has a negative impact on accounting information. That is, only after the number of people affected by the epidemic has changed for more than 3 days does society correct the institutional environment of the epidemic.

#### Accounting Transparency and Fear Sentiment

We find that an increase in accounting transparency can drive COVID-19 searches by Google. In particular, accounting transparency has a positive impact on the increase in the search volume on COVID-19 within a three-period lag. After the three-period lag, the number of new epidemics has a positive impact on accounting information.

#### Fear Sentiment and COVID-19

As expected, we find that an increase in fear sentiment can be driven by fear of COVID-19, as indicated by a significant and positive relationship between fear sentiment and COVID-19. Our results are consistent with those of Baig et al. (45) and Chen et al. (11).

### Robustness Test

Robustness testing is mainly achieved in two ways. On the one hand, by replacing global epidemics with the United States, the final results are shown in Table 6, which is consistent with the results above. On the other hand, we changed the opacity index instrumental variable to the corruption perceptions index. Through the data from Japan, Korea, Spain, the United Kingdom, Australia, France, Singapore, Italy, Germany, and Canada, we obtained Table 7. It can be seen that the impact of COVID-19 is ahead of fear sentiment, while the lag order generally increases as accounting transparency decreases. The result is also consistent with the above empirical results.

**Table 6:** VAR estimations for accounting transparency, fear sentiment, and USA daily new patient.

	**Coef**.	**Std. Err**.	***z***	***P* > *z***	**(95%Conf. Interval)**	**Interval**
**lnSearchpopularity**						
L1.	−0.053	0.007	−7.330	0.000	−0.067	−0.039
L2.	0.052	0.007	7.660	0.000	0.039	0.065
**dlnUSA**						
L1.	−0.001	0.002	−0.420	0.672	−0.004	0.003
L2.	0.003	0.002	1.610	0.108	−0.001	0.006
_cons	0.004	0.007	0.570	0.570	−0.010	0.018
**lnSearchpopularity**						
**Dlnusaopacity**						
L1.	0.084	0.171	0.490	0.622	−0.251	0.420
L2.	0.392	0.171	2.290	0.022	0.056	0.727
**dlnUSA**						
L1.	0.006	0.008	0.700	0.486	−0.010	0.022
L2.	−0.002	0.008	−0.220	0.829	−0.018	0.014
_cons	0.241	0.034	7.180	0.000	0.175	0.307
**dlnUSA**						
**Dlnusaopacity**						
L1.	−1.002	0.761	−1.320	0.188	−2.494	0.489
L2.	−0.378	0.761	−0.500	0.620	−1.870	1.114
**lnSearchpopularity**						
L1.	0.294	0.154	1.910	0.056	−0.008	0.595
L2.	−0.395	0.145	−2.720	0.006	−0.679	−0.111
_cons	0.416	0.149	2.780	0.005	0.123	0.708

**Table 7:** Country-based robustness test.

**Country**	**Score**	**Rank**	**COVID-19** ** → Fear**		**Fear** ** → COVID-19**	
			**Lag**	**Value & *P***	**Lag**	**Value & *P***
Singapore	85	3	L2.	0.0177 (0.008)	L3.	0.5536 (0.065)
Germany	80	9	L3.	0.0082 (0.113)	L4.	−0.6525 (0.011)
Canada	77	11	L1.	0.0127 (0.042)	L1.	0.4879 (0.018)
UK	77	11	L2.	0.0252 (0.028)	L1.	−0.4712 (0.000)
Australia	76	15	L1	−0.0108 (0.066)	L1.	−0.6648 (0.000)
Japan	74	19	L2.	0.0654 (0.070)	L1.	0.4814 (0.042)
France	69	23	L3.	0.0138 (0.002)	L4.	−0.3828 (0.225)
USA	67	25	L3.	0.0359 (0.000)	L4.	−0.3931 (0.005)
Korea	62	32	L4.	0.0285 (0.005)	L1.	−0.2932 (0.016)
Spain	62	32	L2.	0.0654 (0.070)	L1.	0.4815 (0.042)
Italy	53	52	L4.	0.0207 (0.024)	L4.	−0.3811 (0.001)

## Further Discussion: Organization of Public Health Security and An Early Warning System

The above research conclusions provide a basis for us to analyze the institutional environment and individual responses during the epidemic. In practice, it is also necessary to pay attention to the logical basis for the construction of an early warning system for public health security.

If a series system is composed of *n* units of the same type, each unit has its lifetime *X*_*i*_,*i* = 1, 2, …, *n*, where *X*_*i*_ with common distribution function *F* and survival function$\bar{F}=1-F$. Then, the series system has a lifetime*X*_*s*_ = min{*x*_1_, *x*_2_ … *x*_*n*_}; hence, the survival function of *X*_*s*_ is $\bar{F_{S}}={\bar{F_{S}}}^{n}$. Similarly, the lifetime of a parallel system composed of *n* such units is *X*_*p*_ = max{*x*_1_, *x*_2_ … *x*_*n*_}, and the survival function of the parallel system is $\bar{F_{p}}=1-F^{n}$. Evidently, the reliability of the series system $\bar{F_{S}}$ is decreasing in *n*, but the cost of the system is increasing in *n*. For a parallel system, its reliability $\bar{F_{p}}$ is increasing in *n*, but its cost is also increasing in *n*. Therefore, a series system is a bad system, while a parallel system is a good system. For a system composed of some units, if the units have a kind of property, we conclude that the system also has this property, then we say that this system has the closure property for this property. Conversely, if a system is of some property, we conclude that the units are also of some property, we conclude that the units are also of the same property by the structure of the system, then we say that this system is of the reversed property for this property.

Let *X* and *Y* be two non-negative random variables representing two random lifetimes or two risk assets *X* with the probability density function *f*_*x*_, the distribution function *F*_*x*_, the survival function $\bar{F_{x}}$, and the quautile function $F_{x}^{-1}$, respectively. Similarly, we denote *g*_*y*_, *G*_*y*_, $\bar{G_{y}}$ and $G_{y}^{-1}$, respectively, for *Y*. For two random variables *x* and *y* and a partial relation ≤ 0, if *x* ≤ *y*, we say that *y* controls *x*, or we say that *x* is controlled by *y*. Lei et al. (46) defined the DCPQE order, they studied the closure and reversed closure properties of the DCPQE order with respect to the series system and parallel system, risk aversion and risk preference transforms, respectively. Kang and Yan (47) defined the DCRQE order, and they studied some closure and reversed closure properties of this stochastic order with respect to the series and parallel system, respectively. The DCPQE and DCRQE orders are a pair of dual stochastic orders.

## Discussion

This study proposes the logical system and practical measures of a public health security early warning system. On the one hand, the specific mechanism of accounting information transparency for panic and epidemic prevention and control is analyzed at an empirical level. On the other hand, according to the idea of matching, the organization of the early warning system is further analyzed. The results show that two proxy variables, accounting transparency and fear sentiment, are appropriate, and the negative relationship between them has significance for epidemic prevention and control. Moreover, this logical relationship inspires us to use individuals' emotional characteristics to construct a public health security early warning system and adopt a parallel system to achieve better epidemic prevention and control. Compared with previous studies on panic and epidemic prevention and control, such as Chen et al.'s research on the relationship between fear sentiment and bitcoin price (11), our results have more practical significance in epidemic prevention. Compared with articles about epidemic prevention and control systems, such as Jongejan et al.'s research on safety chains (26), the conclusions from this study emphasize the significance of the logical relationship between institutional construction and individual emotions.

Drawing on the immutable characteristics of blockchain technology, blockchain technology was used to record the production, sales and consumption process, market structure, and intellectual property protection in the public health security early warning system. The implications of the findings are as follows: First, fear sentiments about the epidemic are an unavoidable variable in public health. Using the value judgments of public health participants can aid in the formation of the epidemic reporting system's initiator and responsible person. Second, the public health security early warning system can be adopted as a parallel system. Starting from medical, business, logistics, health, and epidemic prevention channels, the blockade of information islands can be broken with the focus on the early warning system, with clear rights and responsibilities. Third, the public health security early warning system based on the parallel system is conducive to building a new pattern of national public health security governance.

More specifically, the blockchain database can be used to record the production, sales and consumption processes, market structure, and intellectual property protection. For this reason, the conclusions of this study include: first, the value judgement of public health participants helps to form the initiators and responsible persons of the epidemic reporting system. Second, the construction of a public health early warning system can break the silos and form clear rights and responsibilities that focus on the overall situation. Third, through the five important components, including people, things, information, the environment, and technology, and effective public health security early warning system can be built. Fourth, blockchain technology is conducive to building a new pattern of value distribution and improving the country's public health security governance capabilities.

## Data Availability Statement

Publicly available datasets were analyzed in this study. This data can be found here: https://www.wind.com.cn/NewSite/wft.html.

## Author Contributions

HW wrote the paper and estimations. DK, LY, and JG wrote the paper and methodology. HL reviewed the paper and collected the data. MS wrote the paper and validation. All authors contributed to the article and approved the submitted version.

## Funding

This paper was supported by the National Natural Social Science Foundation of China (Grant No. 21BGL228), Gansu Province Soft Science Project (20CX4ZA023), Gansu Provincial Health Industry Scientific Research Project (GSWSKY2020-54), and the scientific research and innovation team of Zhejiang Wanli University (Grant No. 202036).

## Conflict of Interest

The authors declare that the research was conducted in the absence of any commercial or financial relationships that could be construed as a potential conflict of interest.

## Publisher's Note

All claims expressed in this article are solely those of the authors and do not necessarily represent those of their affiliated organizations, or those of the publisher, the editors and the reviewers. Any product that may be evaluated in this article, or claim that may be made by its manufacturer, is not guaranteed or endorsed by the publisher.
